# Chilblains Treated with Nitrate of Silver

**Published:** 1846-01

**Authors:** 


*Chilblains treated with Nitrate of Silver.*—Dr. Lauer, chief surgeon in the Kaiser Alexander grenadier regiment, in Berlin, is of opinion, that, in chilblains, no matter whether ulcerated or merely in the state of inflammation, the application of lapis infernalis (fused nitrate of silver) is the most valuable remedy; and he unhesitatingly recommends this treatment as the most applicable. It effects the cure with the greatest certainty, and in the shortest time, and, by restoring the altered texture of the inflamed parts to the normal state, it is, at the same time, the surest means for preventing relapses, so common in chilblains.

The treatment is as follows:—Simple chilblains are first a little moistened, then slightly touched all over their surface by the nitrate. Broken chilblains are strongly cauterized, and the humid places afterwards allowed to dry in the air. As soon as pus is gathering under the cuticle, it is removed by pressure, and then, with a well-pointed piece of nitrate, the spot under the cuticle is cauterized again. When the whole is dry, the ulcer may be considered cured the moment the cuticle falls off. Sometimes, rather deep and large chilblains are cured by one single cauterization; sometimes two or three; and rarely more applications are requisite. The unpleasant itching disappears soon after the cauterization.—*Med. Times*, from *Med. Zeit.*, May 14, 1845, in *Med. News.*

